# Effects of Modulating BMP9, BMPR2, and AQP1 on BMP Signaling in Human Pulmonary Microvascular Endothelial Cells

**DOI:** 10.3390/ijms25158043

**Published:** 2024-07-24

**Authors:** Nikolaos S. Lotsios, Chrysi Keskinidou, Ioanna Dimopoulou, Anastasia Kotanidou, David Langleben, Stylianos E. Orfanos, Alice G. Vassiliou

**Affiliations:** 1 First Department of Critical Care Medicine & Pulmonary Services, School of Medicine, National and Kapodistrian University of Athens, Evangelismos Hospital, 106 76 Athens, Greece; n.lotsios96@gmail.com (N.S.L.); chrysakes29@gmail.com (C.K.); idimo@otenet.gr (I.D.); akotanid@med.uoa.gr (A.K.); 2 Center for Pulmonary Vascular Disease, Azrieli Heart Center and Lady Davis Institute, Jewish General Hospital, McGill University, Montreal, QC H3T 1E2, Canada; david.langleben@mcgill.ca

**Keywords:** PAH, BMP9, BMPR2, AQP1

## Abstract

Pulmonary arterial hypertension (PAH) is a chronic disease characterized by a progressive increase in mean pulmonary arterial pressure. Mutations in the *BMPR2* and *AQP1* genes have been described in familial PAH. The bone morphogenetic proteins BMP9 and BMP10 bind with high affinity to BMPR2. Administration of BMP9 has been proposed as a potential therapeutic strategy against PAH, although recent conflicting evidence dispute the effect of such a practice. Considering the involvement of the above molecules in PAH onset, progression, and therapeutic value, we examined the effects of modulation of BMP9, BMPR2, and AQP1 on BMP9, BMP10, BMPR2, AQP1, and TGFB1 expression in human pulmonary microvascular endothelial cells in vitro. Our results demonstrated that silencing the *BMPR2* gene resulted in increased expression of its two main ligands, namely BMP9 and BMP10. Exogenous administration of BMP9 caused the return of *BMP10* to basal levels, while it restored the decreased AQP1 protein levels and the decreased TGFB1 mRNA and protein expression levels caused by *BMPR2* silencing. Moreover, *AQP1* gene silencing also resulted in increased expression of BMP9 and BMP10. Our results might possibly imply that the effect of exogenously administered BMP9 on molecules participating in the BMP signaling pathway could depend on the expression levels of *BMPR2*. Taken together, these results may provide insight into the highly complex interactions of the BMP signaling pathway.

## 1. Introduction

Pulmonary hypertension (PH) is characterized as a chronic and progressive disorder [1]. Pulmonary arterial hypertension (PAH), one of the five subgroups of PH, mainly affects the distal pulmonary arteries and occurs in both idiopathic and hereditary forms. Loss and obstructive remodeling of the pulmonary vascular bed are responsible for the progressive increase in pulmonary arterial pressure and pulmonary vascular resistance, leading to right heart failure and eventually death [2,3,4]. PAH onset and development are highly affected by alterations in the signaling pathways governed by members of the transforming growth factor-β (TGF-β) family. Hereditary cases of PAH commonly present a variety of mutations in the gene encoding for the bone morphogenetic protein receptor type II (BMPR2), leading to the protein’s loss of function [5,6,7]. In addition to hereditary PAH, ~10–40% of idiopathic PAH cases carry *BMPR2* mutations [8]. BMPR2 contributes significantly to blood vessel formation and function; disrupted signaling due to decreased BMPR2 expression is also predominant even in PAH cases lacking *BMPR2* mutations [9].

In the canonical BMP signaling pathway, BMPR2 acts as a regulator of gene expression through BMP ligand binding, type I receptor activation, and phosphorylation of specific members of the mothers against decapentaplegic (SMAD) family. BMP9 and BMP10 are the primary ligands of the receptor complex, playing a significant role in homeostasis and vascular development [10]. BMP9 is thought to be the principal ligand for BMPR2. As such, it leads to enhanced BMPR2 signaling which is antiproliferative, and would inhibit the formation of PAH pathology. BMP10 is thought to have similar actions [11]. Heterozygous mutations in the genes encoding for BMP9 and BMP10 have been identified in several PAH cases [5,12,13,14].

Current PAH therapies have improved symptoms and functional capacity, and have reduced the incidence of deteriorations. These include orally active phosphodiesterase-5 inhibitors and soluble guanylate cyclase stimulators to raise abnormally low cyclic guanosine monophosphate (GMP) levels, intravenous, subcutaneous, inhaled, and oral prostanoid analogs or mimetics to raise abnormally low cyclic adenosine monophosphate (AMP) levels, and orally active endothelin receptor blockers to reduce the detrimental effects of increased endothelin-1 levels [15]. Recently, sotatercept, an activin-binding molecule, has also shown significant efficacy and has been approved for clinical use. It addresses pulmonary microvascular cell proliferation rather than vascular tone, and it offers the potential to at least partially correct the BMPR2/TGFB signaling imbalance seen in PAH [16]. Although the administration of recombinant BMP9 has been proposed as a novel therapeutic strategy, few experimental studies have explored the important role of BMP9 in the pathogenesis of PAH [11,17,18,19].

Alongside BMP signaling, disrupted TGF-β signaling is central to the development and progression of PAH. TGF-β ligands bind with high affinity to the TβRII/TβRI receptor complex, thus activating the canonical and non-canonical signaling pathways [20]. The TGF-β family has pleiotropic and redundant functions, and it can regulate cellular processes, including cell proliferation, differentiation, and migration [21]. Among the members of the family, growth factor TGF-β1 is of great interest. Specifically, TGF-β1 has a significant role in the synthesis and increased deposition of the extracellular matrix during vascular wall remodeling [22]. Furthermore, it is evident that TGF-β family dysregulation is an important component in PAH, contributing to pulmonary vascular remodeling and inflammation [20].

As stated above, mutations in the *BMPR2* gene and downstream targets of canonical BMP signaling drive PAH development. Recent genetic evidence has identified novel genes with mutations among PAH patients. Mutations in the gene encoding for aquaporin 1 (AQP1) have been described in several cases of hereditary PAH, resulting in the characterization of *AQP1* as a PAH-related gene [5,23]. Aquaporins (AQPs) are membrane channel proteins with a significant role in water transport regulation across the cell membrane [24]. Along with their role in water homeostasis, AQPs contribute to several biological processes, among which are cell proliferation, cell migration, and edema formation [25]. Recently, our group has described a link between AQP1 and members of the BMP signaling pathway, including BMPR2 and TGF-β1 [26,27].

Based on the above, in the present study, we aimed to examine the mRNA and protein expression patterns of BMP9 and BMP10 in human pulmonary microvascular endothelial cells (HPMECs) where we silenced the expression of *AQP1* and *BMPR2*. Considering the therapeutic role of BMP9, we investigated the effect of exogenous BMP9 administration on AQP1, BMPR2, BMP10, and TGFB1 mRNA and protein expression in *BMPR2*-silenced HPMECs, a commonly used in vitro PAH model.

## 2. Results

### 2.1. Effects of Modulating BMPR2 and BMP9 on BMP Signaling Molecules in Human Pulmonary Microvascular Endothelial Cells

**Figure 1:**
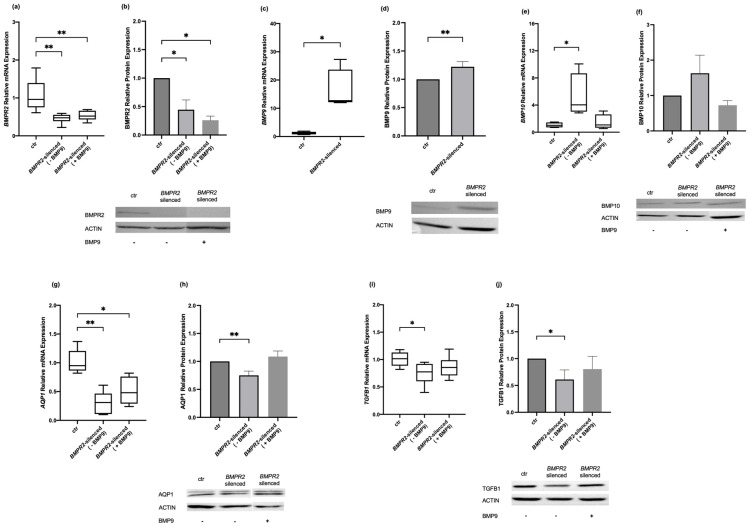
Effects of modulating *BMPR2* and BMP9 on BMP signaling molecules in human pulmonary microvascular endothelial cells. HPMECs were silenced for the *BMPR2* gene, and the relative mRNA and protein expression of BMPR2 (**a**,**b**), BMP9 (**c**,**d**), BMP10 (**e**,**f**), AQP1 (**g**,**h**), and TGFB1 (**i**,**j**) were estimated before and after the exogenous administration of BMP9. Relative mRNA expression is depicted with box plots (line in the middle, median values; box edges, 25th and 75th percentiles; whiskers, range of values). Relative protein expression is depicted with bar plots (mean + SEM). Results from at least three independent experiments are presented. To ensure consistency and reproducibility across the independent experiments, specificity (siRNA negative control), efficiency (*BMPR2* siRNA), and the effect of BMP9 exogenous administration on the non-transfected controls were tested each time. Statistical analysis was performed using the Mann–Whitney test or Student’s *t*-test, as appropriate. *, *p* < 0.05; **, *p* < 0.01 compared to non-transfected control cells.

Considering the proposed role of BMP9 as a potential therapeutic in PAH, we decided to explore the effect of modulating *BMPR2* and BMP9 on molecules implicated in BMP signaling. Initially, we silenced the HPMECs for *BMPR2*. At first, we tested transfection specificity by transfecting the cells with a universal scrambled negative control siRNA duplex (siRNA controls). The resultant *BMPR2* expression levels in the siRNA controls were 0.71 (0.52–1.15) vs. 0.96 (0.75–1.39) in the non-transfected control cells (*p* > 0.05). We then proceeded with the transfection with the *BMPR2*-specific siRNA, which resulted in silencing the *BMPR2* gene by 50% compared to the non-transfected control cells (0.48 (0.39–0.55) vs. 0.96 (0.75–1.39), respectively, *p* < 0.01; Figure 1a). The same effect was observed regarding BMPR2 protein levels compared to the non-transfected control cells (0.45 ± 0.17, *p* < 0.05; Figure 1b). The effect of *BMPR2* silencing was subsequently studied on *BMP9* and *BMP10* mRNA expression. *BMP9* mRNA expression was increased nearly 13-fold compared to the non-transfected control cells (12.69 (12.14–23.67) vs. 1.20 (0.77–1.67), respectively, *p* < 0.05; Figure 1c) and protein expression increased by 22% in the *BMPR2*-silenced cells compared to the non-transfected control cells (1.22 ± 0.09, *p* < 0.01; Figure 1d). *BMP10* mRNA expression increased four-fold compared to the non-transfected control cells (4.03 (3.03–8.68) vs. 1.01 (0.72–1.44), respectively, *p* < 0.05; Figure 1e), whereas BMP10 protein levels remained unaltered compared to the non-transfected control cells (1.69 ± 0.51, *p* > 0.05; Figure 1f).

Exogenous administration of BMP9 to the *BMPR2*-silenced cells did not have any effect on the BMPR2 mRNA and protein expression levels, which remained reduced compared to the non-transfected control cells. The *BMPR2* mRNA levels remained lower than the non-transfected control cells (0.52 (0.43–0.66) vs. 0.96 (0.75–1.39), respectively, *p* < 0.01; Figure 1a), and the same applied to its protein levels (0.26 ± 0.07, *p* < 0.01; Figure 1b). On the other hand, following BMP9 exogenous administration, the expression levels of *BMP10* mRNA returned to its levels prior to treatment and did not differ from the non-transfected control cells (1.07 (0.66–2.62) vs. 1.01 (0.72–1.44), respectively, *p* > 0.05; Figure 1e). BMP10 protein levels, however, were not affected (0.73 ± 0.13, *p* > 0.05; Figure 1f).

Given our previous published results on the effect of *BMPR2* silencing on *AQP1* and *TGFB1* expression [26,27], we then proceeded to examine the effect of BMP9 exogenous administration on AQP1 and TGFB1 mRNA and protein expression in *BMPR2*-silenced HPMECs. After reconfirming our previous findings that *BMPR2* silencing results in decreased *AQP1* expression compared to the non-transfected control cells (0.31 (0.11–0.47) vs. 0.95 (0.87–1.21), respectively, *p* < 0.01; Figure 1g), we examined the effect of BMP9 exogenous administration. No effect of BMP9 on the mRNA expression of *AQP1* was observed, which remained decreased compared to the non-transfected control cells (0.48 (0.29–0.76) vs. 0.95 (0.87–1.21), respectively, *p* < 0.05; Figure 1g). Of most interest, however, exogenous administration of BMP9 could restore the decreased AQP1 protein levels resulting from *BMPR2* silencing. Prior to BMP9 treatment, AQP1 levels decreased to 0.75 ± 0.08 compared to the non-transfected control cells (*p* < 0.01; Figure 1h), whereas after treatment, the lowered levels were restored (1.09 ± 0.10, *p* > 0.05; Figure 1h). Finally, after reconfirming our formerly published results that silencing of *BMPR2* expression results in decreased *TGFB1* mRNA expression compared to the non-transfected control cells (0.78 (0.60–0.92) vs. 1.02 (0.89–1.13), respectively, *p* < 0.05; Figure 1i), we demonstrated that the exogenous administration of BMP9 could restore the *TGFB1* mRNA levels in *BMPR2*-silenced HPEMCs, returning them to levels comparable with the non-transfected control cells (0.86 (0.71–0.99) vs. 1.02 (0.89–1.13), respectively, *p* > 0.05; Figure 1i). We finally examined TGFB1 protein levels in the *BMPR2*-silenced HPMECs. TGFB1 protein levels decreased in the *BMPR2*-silenced cells compared to the non-transfected control cells (0.61 ± 0.18, *p* < 0.05; Figure 1j). The exogenous administration of BMP9 reversed the effect of *BMPR2* silencing on TGFB1 protein levels (0.80 ± 0.24, *p* > 0.05; Figure 1j).

### 2.2. Effects of Modulating AQP1 on BMP Signaling Molecules in Human Pulmonary Microvascular Endothelial Cells

**Figure 2:**
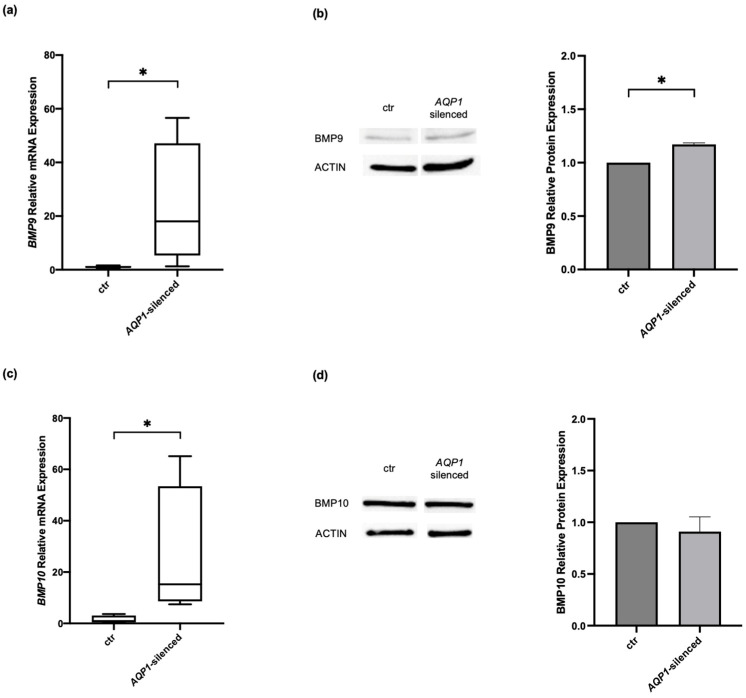
Effects of modulating *AQP1* on BMP signaling molecules in human pulmonary microvascular endothelial cells. HPMECs were silenced for the *AQP1* gene, and the relative mRNA and protein expression of BMP9 (**a**,**b**) and BMP10 (**c**,**d**) were estimated. Relative mRNA expression is depicted with box plots (line in the middle, median values; box edges, 25th and 75th percentiles; whiskers, range of values). Relative protein expression is depicted with bar plots (mean + SEM). Results from at least three independent experiments are presented. To ensure consistency and reproducibility across the independent experiments, specificity (siRNA negative control) and efficiency (*AQP1* siRNA) were tested each time. Statistical analysis was performed using the Mann–Whitney test or Student’s *t*-test, as appropriate. *, *p* < 0.05 compared to the non-transfected control cells.

Our group has previously described the interplay between AQP1 and BMPR2 [26,27]. Considering our previously published data, the finding presented above that BMP9 restores AQP1 protein levels in *BMPR2*-silenced cells, and the fact that BMP9 and BMP10 are the two most important ligands of BMPR2, we next aimed to examine the mRNA and protein expression of these molecules in *AQP1*-silenced HPMECs (Figure 2). As in *BMPR2*, transfection specificity was initially tested by transfecting the cells with a universal scrambled negative control siRNA duplex. The resultant *AQP1* levels in the siRNA controls were 0.90 (0.63–1.77) vs. 1.03 (0.72–1.40) in the non-transfected control cells (*p* > 0.05). Transfection with the *AQP1*-specific siRNA resulted in silencing the *AQP1* gene by 70% compared to the non-transfected control cells (0.30 (0.13–0.53) vs. 1.03 (0.72–1.40), respectively, *p* < 0.05). The *AQP1*-silenced cells showed an increase in both mRNA and protein levels of BMP9 (Figure 2a,b). Specifically, *BMP9* mRNA expression increased 18-fold compared to the non-transfected control cells (18.04 (5.30–47.14) vs. 1.03 (0.71–1.41), respectively, *p* < 0.05; Figure 2a). BMP9 protein levels increased by 17% following *AQP1*-silencing compared to the non-transfected control cells (1.17 ± 0.01, *p* < 0.05; Figure 2b). On the other hand, only the mRNA levels of *BMP10* were found to be significantly altered in *AQP1*-silenced cells compared to the non-transfected control cells (15.23 (8.61–53.42) vs. 0.92 (0.44–3.05), respectively, *p* < 0.05; Figure 2c). BMP10 protein levels remained unaltered (0.91 ± 0.14, *p* > 0.05; Figure 2d).

**Figure 3:**
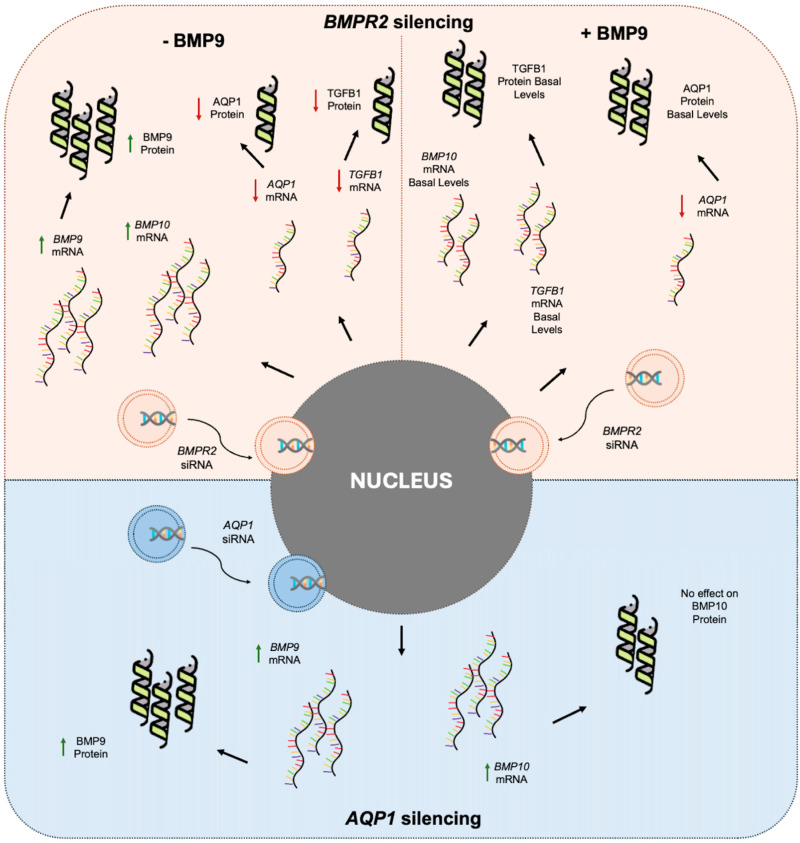
Main findings of the study. In the upper panel (Pink), the mRNA and protein levels of BMP signaling molecules are depicted in human pulmonary microvascular endothelial cells (HPMECs) silenced for *BMPR2* before and after exogenous BMP9 administration. As depicted in the left upper panel, *BMP9* and *BMP10* mRNA levels increased, while *AQP1* and *TGFB1* mRNA levels decreased in the *BMPR2*-silenced HPEMCs prior to BMP9 administration. Protein levels of BMP9 increased, while AQP1 and TGFB1 protein levels decreased. As shown in the upper right panel, following exogenous BMP9 administration, *BMP10* and *TGFB1* mRNA returned to basal levels, while *AQP1* remained downregulated. The AQP1 and TGFB1 proteins returned to basal levels. In the lower panel (Blue), the mRNA and protein levels of BMP9 and BMP10 are depicted following *AQP1* silencing in HPMECs. BMP9 increased at both mRNA and protein levels, while only mRNA levels of *BMP10* were increased. AQP1, aquaporin 1; BMP9, bone morphogenetic protein 9; BMP10, bone morphogenetic protein 10; BMPR2, bone morphogenetic protein receptor type 2; TGFB1, transforming growth factor beta 1. Green arrows represent increased levels and red arrows represent decreased levels. Created with icons by https://www.freepik.com (accessed on 18 July 2024) (dna_268497 and protein_1951419).

In Figure 3, the findings of our study are depicted schematically.

## 3. Discussion

It is well established that dysregulation of the BMP signaling pathway contributes to PAH onset. In this study, we aimed to explore the interplay between the main ligands of BMPR2 and several molecules with a central role in PAH development and progression. We demonstrated that silencing of *AQP1* and *BMPR2* in HPMECs positively affects the expression of both BMP9 and BMP10. We also examined the effect of exogenous BMP9 administration on AQP1, TGFB1, and BMP10 mRNA and protein expression in *BMPR2*-silenced HPEMCs. We observed that the exogenous administration of BMP9 could restore the decreased AQP1 protein levels and the decreased TGFB1 mRNA and protein expression levels caused by *BMPR2* silencing. Finally, our results may possibly imply that in pulmonary endothelial cells, the effect of exogenous BMP9 administration on BMPR2, BMP10, AQP1, and TGFB1 expression might be dependent on the expression levels of *BMPR2*. The mechanism, however, is unclear. BMP9 acts mainly as the ligand for BMPR2, so BMPR2 levels might be critical to BMP9 signaling.

Acknowledging the importance of endothelial cells in PAH, our group has demonstrated a possible interplay between AQP1 and several members of the BMP signaling pathway in HPMECs. More specifically, silencing of *AQP1* resulted in decreased *BMPR2* mRNA, whereas *BMPR2*-silenced HPMECs presented downregulation of AQP1 mRNA, protein expression, and function [26,27]. Aiming to further explore this interaction, we chose to examine the expression patterns of the BMPR2 ligands, BMP9 and BMP10, in *AQP1*- and *BMPR2*-silenced HPMECs. In both cases, silencing of either *AQP1* or *BMPR2* resulted in the mRNA upregulation of both ligands and the increase in BMP9 protein expression. These results further confirm the complex interplay between AQP1 and BMPR2, although they raise novel questions regarding the order in which these changes in expression levels take place.

BMP9 is regarded as the main ligand of BMPR2, and its pro- and anti-angiogenic properties have been at the epicenter of a discourse most relevant in PAH. Most importantly, findings regarding the role of BMP9 in PAH have been conflicting. In several studies, BMP9 has been proposed to promote pulmonary arterial remodeling. Tu and colleagues showed that *BMP9*-knock-out mice exposed to chronic hypoxia demonstrate decreased endothelin-1 and increased apelin and adrenomedullin mRNA levels compared to wild-type *BMP9* mice, thus proposing that selective inhibition of BMP9 in mice could prevent the development of experimental pulmonary hypertension [19]. Furthermore, the increase in wall thickness and muscularization of distal pulmonary arteries under hypoxic conditions was shown to be repressed by single *BMP9* or double *BMP9*/*BMP10* deletions [28]. Pathologic variants in *GDF2*, the gene for BMP9 are known to cause typical PAH [29,30,31]. In a recent publication, Theilmann and colleagues showed that BMP9 administration increased proliferation in blood outgrowth endothelial cells (BOECs) derived from PAH patients bearing *BMPR2* mutations, as well as in *BMPR2*-silenced human pulmonary artery endothelial cells. Most importantly, using conditional knockout mice, the authors correlated *BMPR2* expression and endothelial proliferation. A reduction greater than 50% was needed for proliferation to be induced [11].

In our HPMECs, *BMPR2* silencing resulted in a greater than 50% reduction in *BMPR2* mRNA expression. This reduction in *BMPR2* gene expression was accompanied by enhanced BMP signaling, evident by the mRNA increase in both *BMP9* and *BMP10*, and the protein increase in BMP9. Administration of BMP9 returned *BMP10* mRNA to control levels, depicting the presence of a possible equilibrium between BMP9 and BMP10 levels. Furthermore, we observed that the downregulation of ΤGFΒ1 mRNA and protein caused by *BMPR2* silencing was seemingly reversed by BMP9 administration. This could possibly lead to enhanced TGFβ-signaling, a known hallmark of PAH [32]. On a functional level, it has been accepted that reprogramming of endothelial cells through endothelial-to-mesenchymal transition (EndMT) retains a role in the progression of PAH. Interestingly, BMP9 administration to pulmonary microvascular endothelial cells (MVECs) derived from PAH patients impaired the endothelial barrier function [18,33].

On the other hand, compromised BMP9 expression is reported in clinical PAH cases. As previously noted, *BMP9* gene mutations have been associated with PAH. Plasma BMP9 levels were reportedly reduced in female PAH patients carrying mutations in the *BMP9* gene. The presence of *BMP9* mutations hampered BMP10 plasma levels as well [34]. Circulating BMP9 has been reported to be undetectable in several studies focusing on pediatric PAH patients carrying mutations in the *BMP9* gene. In some cases, BMP10 levels were found to be reduced as well [30,31,35]. Of note, circulating BMP9 levels were found markedly reduced in patients with portopulmonary hypertension (PoPH), in comparison to healthy controls. A decrease in circulating BMP9 was also confirmed in CCl4 mice. Most importantly, low BMP9 levels were shown to dispose a significant ability in predicting the development of PoPH in patients with liver disease [36].

Recently, BMP9 treatment has been reported to affect BMPR2 expression. In HPAECs silenced for *ALK1*, the BMPR2 gene and protein expression has been shown to increase following BMP9 treatment [37]. Similarly, Long et al. demonstrated an increase in BMPR2 mRNA and protein expression in PAECs post-BMP9 administration [17]. In our series of experiments, BMP9 failed to return *BMPR2* mRNA to baseline levels in *BMPR2*-silenced HPMECs. A similar finding to ours was described by Upton and colleagues in HPAECs, demonstrating that the effect of BMP9 administration on BMPR2 expression is affected by the underlying *BMPR2* expression levels [37]. Long and colleagues further examined the therapeutic value of BMP9 use in PAH demonstrating its ability to reverse PAH pathology in both genetic and non-genetic animal models. The effect of BMP9 administration on PAH pathophysiology could possibly be correlated to existing BMPR2 expression levels. Administration of BMP9 proves to be successful in attenuating apoptosis in BOECs from both healthy controls and PAH patients carrying *BMPR2* mutations. Interestingly, its anti-apoptotic effect was ameliorated in PAECs previously silenced for *BMPR2* expression [17].

As previously stated, AQP1 has been suggested to have a potentially causative role in PAH, with rare missense variants being overrepresented in patients with familial PAH [5]. AQPs’ diverse roles range from cell proliferation and migration, angiogenesis, inflammatory responses, and signaling transduction [38,39]. Regarding AQP1 expression, Li and colleagues have demonstrated that treatment with pro-BMP9 resulted in a significant decrease in *AQP1* mRNA expression in HPEMCs [40]. Interestingly, in our series of experiments performed in *BMPR2*-silenced HPMECs, even though BMP9 treatment had no effect on the decreased *AQP1* mRNA expression, it did cause AQP1 protein expression to return to pre-treatment levels.

Our results, in combination with existing evidence in the published literature, could propose that the effects of exogenous BMP9 administration are closely dependent on *BMPR2* expression levels and could grant a greater perspective on the interaction between BMP signaling and AQP1 expression. Our results offer a glimpse at the complexity of receptor-ligand signaling in PAH, and suggest that further studies are warranted.

Among the limitations of our study is the fact that we only utilized a single in vitro cell model in our experiments. Further investigation on animal models as well as cell samples derived from PAH patients could prove to be beneficial. In addition, our study focused on the genetic and protein expression patterns of the studied molecules. In the present study, we focused on the pathophysiology of PAH and mainly on the genetic and protein expression patterns of studied molecules. The precapillary pulmonary microvasculature, where PAH develops, is a unique circulation in the body, with different embryologic origin and different vasodilator/constrictor responses as compared to the systemic circulation. Future studies could focus on revealing the functional and mechanistic effects of BMP9 administration on *BMPR2*-silenced cells. Finally, the therapeutic implications of BMP9 administration should be further investigated in clinical studies, which provide concrete data on clinical benefit. On the other hand, a strong aspect of our study is that it explores in greater depths the interplay between AQP1 and BMPR2, two known mediators in PAH onset. To the best of our knowledge, this is the first study to demonstrate the effect of BMP9 administration on AQP1 expression in the setting of reduced *BMPR2* expression, thus shedding light onto the complex interplay between BMP signaling and AQP1 expression.

## 4. Materials and Methods

### 4.1. Cell Culture

The designed experiments were performed using the HPMEC-ST1.6R cell line, which was generated from human pulmonary microvascular endothelial cells isolated from an adult donor (male, 63 years old, malignant tumor, passage 24). In our case, the cell line was a kind gift from Dr. Donald E. Unger of the Johannes Gutenberg-Universität in Mainz [41]. Cells were incubated at 37 °C with stable 5% CO_2_ levels. M199 medium (Biosera, Cholet, France) supplemented with 20% fetal bovine serum (PAN-Biotech, Aidenbach, Germany), 1% L-glutamine, 1% antibiotics (Biowest, Nuaille, France), 25 μg/mL heparin (LEO Pharmaceutical Hellas S.A., Chalandri, Greece), and 50 μg/mL endothelial cell growth serum (MilliporeSigma, Burlington, MA, USA) was used for cell culture.

### 4.2. Transfection in Human Pulmonary Microvascular Endothelial Cells

The transfection assay was performed according to the manufacturer’s instructions using the SiTran2.0 transfection reagent supplied by OriGene (OriGene, Rockville, MD, USA). The day prior to transfection, the cells were plated to achieve 50–70% confluency on transfection day. On the day of transfection, 5.5 nM of the siRNA (OriGene) were added to the cells in serum-free medium (Opti-MEM, Thermo Fisher Scientific, Waltham, MA, USA). Subsequently, 5 h post-transfection, the serum-free medium containing the siTran/siRNA mixture was replaced with fully supplemented M199 medium (Biosera, Cholet, France).

### 4.3. BMP9 Treatment of Human Pulmonary Microvascular Endothelial Cells

Cells were treated with BMP9 (OriGene, Rockville, MD, USA) 24 h post-transfection. BMP9 was added to fully supplemented M199 medium to a final concentration of 5 ng/mL. The cells were incubated at 37 °C and 5% CO_2_ for 24 h, and were subsequently harvested for total RNA and protein extraction.

### 4.4. RNA Extraction

Total RNA was extracted using the TRI reagent (Thermo Fisher Scientific, Waltham, MA, USA) according to the manufacturer’s instructions. A spectrophotometer was used to assess the concentration and quality of isolated total RNA at 260/280 nm, and RNA integrity was evaluated with formaldehyde agarose gel electrophoresis.

### 4.5. Reverse Transcription and Quantitative Real-Time PCR

From each sample, 100 ng of total RNA were used to synthesize cDNA (Nippon Genetics, Duren, Germany) following the manufacturer’s instructions. Subsequently, the quantitative real-time polymerase chain reaction (qPCR) method (Kapa SYBR^®^ Green PCR Master Mix, Sigma-Aldrich, St Louis, MO, USA) was performed to measure *AQP1*, *BMP9*, *BMP10*, *BMPR2*, *TGFB1*, and *GAPDH* mRNA expression. The analysis was performed on a CFX Connect thermocycler (Bio-Rad Laboratories, Inc., Hercules, CA, USA). In Table 1, the specific primer set used for each gene is listed.

The comparative CT method 2^−ΔΔCT^ was used for the calculation of relative gene expression levels of treated cells versus untreated control cells [42]. *GAPDH* mRNA expression levels were used for normalization purposes.

### 4.6. Protein Determination

Cells were homogenized on ice using a handheld homogenizer, following the addition of lysis buffer consisting of 250 mM sucrose, 10 mM Tris-HCl, 10 mM NaCl, 1 mM EDTA, 0.1 mM PMSF, and 1% *v*/*v* Triton-X 100, with a pH 7.4. The homogenate was centrifuged at 1000× *g* at 4 °C for 5 min. Following centrifugation, the supernatant containing the extracted proteins was collected and stored at −80 °C until used. The bicinchoninic acid (BCA, Thermo Fisher Scientific, Waltham, MA, USA) method was used for the determination of total protein concentration [43]. Bovine serum albumin was used as the standard.

### 4.7. SDS-Polyacrylamide Gel Electrophoresis (PAGE) and Immunoblotting of Human Pulmonary Microvascular Endothelial Cells’ Homogenates

A “Biorad Mini Protean II” electrophoresis apparatus (Bio-Rad Laboratories, Inc., Hercules, CA, USA) and 12% or 15% polyacrylamide slab gels were utilized to perform SDS-PAGE, as previously described [44]. A wet transfer apparatus (Bio-Rad Laboratories, Inc., Hercules, CA, USA) allowed for the western transfer onto an Immobilon-P PVDF membrane (0.45 L pore size, MilliporeSigma, Burlington, MA, USA) [45]. Immunological detection followed using specific antibodies for AQP1 (MilliporeSigma, Burlington, MA, USA), BMP9 (Elabscience, Houston, TX, USA), BMP10 (Affinity Biosciences, Cincinnati, OH, USA), BMPR2 (Affinity Biosciences, Cincinnati, OH, USA), and TGFB1 (Affinity Biosciences, Cincinnati, OH, USA). Actin (MilliporeSigma, Burlington, MA, USA) was used as loading controls. Enhanced chemiluminescence (ECL) was performed to detect proteins (MilliporeSigma, Burlington, MA, USA). Densitometry was applied to estimate relative protein expression using the Thermo Fisher iBright CL1500 Imaging System (Thermo Fisher Scientific, Waltham, MA, USA).

### 4.8. Statistical Analysis

Data are depicted as box plots or bar plots, presenting median values with interquartile range (IQR) (skewed distribution) or mean + SEM (normal distribution). Statistical analysis was performed using the non-parametric Mann–Whitney test, or Student’s *t*-test, as appropriate. All tests were performed using the GraphPad Prism 9 software (GraphPad Software, San Diego, CA, USA). All *p*-values are two-sided; statistical significance was set at *p* < 0.05.

## 5. Conclusions

We have previously shown the interaction between the *BMPR2* and *AQP1* genes. Herein, we further explored this relationship and were able to expand this knowledge by demonstrating the effect of *AQP1* expression levels on the expression of the ligands of BMPR2, namely BMP9 and BMP10. Furthermore, we demonstrated that silencing the *BMPR2* gene resulted in increased expression of BMP9 and BMP10. Exogenous administration of BMP9 caused a return in BMP10 to basal levels, while it restored the decreased AQP1 protein levels and the decreased TGFB1 mRNA and protein expression levels caused by *BMPR2* silencing. Our results might possibly imply that the effect of exogenously administered BMP9 on molecules participating in the BMP signaling pathway could depend on the expression levels of *BMPR2*. In conclusion, our results provide a new perspective into the complex interactions of BMP signaling. Further experiments in animal models and the clinical setting could provide a better understanding of the underlying mechanisms that govern PAH and its therapeutic interventions.

## Figures and Tables

**Table 1:** Gene-specific primer pairs used in quantitative real-time PCR experiments.

Gene		Sequence (5′-3′)	nt
*AQP1*	F	5′-TATGCGTGCTGGCTACTACCGA-3′	22
	R	5′-GGTTAATCCCACAGCCAGTGTAG-3′	23
*BMP9*	F	5′-CCTGCCCTTCTTTGTTGTCTTCTC-3′	24
	R	5′-TGACTGCTCTCACCTGCCTCTGTG-3′	24
*BMP10*	F	5′-AAGCCTATGAATGCCGTGGTG-3′	21
	R	5′-AGGCCTGGATAATTGCATGCTT-3′	20
*BMPR2*	F	5′-CCACCTCCTGACACAACACC-3′	20
	R	5′-TGTGAAGACCTTGTTTACGGT-3′	21
*GAPDH*	F	5′-ATGGGGAAGGTGAAGGTCG-3′	19
	R	5′-GGGGTCATTGATGGCAACAATA-3′	22
*TGFB1*	F	5′-GCGTGCTAATGGTGGAAAC-3′	19
	R	5′-CGGTGACATCAAAAGATAACCAC-3′	23

*AQP1*, aquaporin 1; *BMP9*, bone morphogenetic protein 9; *BMP10*, bone morphogenetic protein 10; *BMPR2*, bone morphogenetic protein receptor type 2; *GAPDH*, glyceraldehyde-3-phosphate dehydrogenase; *TGFB1*; transforming growth factor beta 1.

## Data Availability

Data are contained within the article.
